# Antibody fragments structurally enable a drug-discovery campaign on the cancer target Mcl-1

**DOI:** 10.1107/S2059798319014116

**Published:** 2019-10-31

**Authors:** Jakub Luptak, Michal Bista, David Fisher, Liz Flavell, Ning Gao, Kate Wickson, Steven L. Kazmirski, Tina Howard, Philip B. Rawlins, David Hargreaves

**Affiliations:** a Discovery Sciences, R&D Biopharmaceuticals, AstraZeneca, Cambridge CB4 0WG, England; b Discovery Sciences, R&D Biopharmaceuticals, AstraZeneca, Waltham, MA 02451, USA

**Keywords:** Mcl-1, scFv, Fab, drug design

## Abstract

Apoptosis is a crucial process by which multicellular organisms control tissue growth, removal and inflammation. Disruption of the normal apoptotic function is often observed in cancer, where cell death is avoided by the overexpression of anti-apoptotic proteins of the Bcl-2 (B-cell lymphoma 2) family, including Mcl-1 (myeloid cell leukaemia 1). This makes Mcl-1 a potential target for drug therapy, through which normal apoptosis may be restored by inhibiting the protective function of Mcl-1. Here, the discovery and biophysical properties of an anti-Mcl-1 antibody fragment are described and the utility of both the scFv and Fab are demonstrated in generating an Mcl-1 crystal system amenable to iterative structure-guided drug design.

## Abstract 2

Mcl-1 is an important cancer target for drug therapy, through which normal apoptosis may be restored by inhibiting its protective function. An scFv and a Fab have been used to generate an apo Mcl-1 crystal system that is amenable to iterative structure-guided drug design.

## 1. Introduction   

Mcl-1 (myeloid cell leukaemia 1) is a member of the Bcl-2 (B-cell lymphoma 2) family of pro-survival proteins that regulate programmed cell death via apoptosis (Elmore, 2007 ▸). It comprises a C-terminal transmembrane helix anchor by which it associates with the mitochondrial membrane, an eight-helix bundle responsible for signalling via protein–protein interaction and a mostly disordered N-terminal region that includes a PEST-like domain (Day *et al.*, 2004 ▸). Intrinsic or extrinsic cell signals are received through a complex cascade which eventually affect the balance of pro- and anti-apoptotic protein complexes at the mitochondrial membrane. The final effect is the permeation of the mitochondrial membrane, with the release of cytochrome *c* into the cytoplasm, resulting in cell death. Mcl-1 is an anti-apoptotic member of the Bcl-2 family which binds to pro-apoptotic proteins containing the BH3 domain, a short α-helical stretch of around 16 residues. Binding of Mcl-1 to the pro-apoptotic proteins prevents apoptosis. Normally, this process is finely regulated, but in certain types of cancers this regulation breaks down. A therapeutic approach has been pursued to bind and inhibit Mcl-1, promoting apoptosis in diseased tissues (Lee *et al.*, 2008 ▸; Bruncko *et al.*, 2015 ▸; Kotschy *et al.*, 2016 ▸; Johannes *et al.*, 2017 ▸; Tron *et al.*, 2018 ▸).

Some of the BH3 helices from proteins such as Bim, Bid and Noxa have been crystallized with Mcl-1, revealing a common binding mode for the helix-mediated protein–protein interaction in a long, shallow hydrophobic groove (Czabotar *et al.*, 2007 ▸). It is the nature of this binding site that makes finding specific, highly potent peptidomimetics so challenging for drug discovery. Furthermore, the combination of a large surface-exposed binding groove and a small overall protein size makes the crystallization of Mcl-1 ligand dependent. It has been observed that crystal contacts are mediated through the ligand or, in some cases, through ligand–ligand interactions (Clifton *et al.*, 2015 ▸; Tron *et al.*, 2018 ▸).

Macromolecular crystallography plays a crucial role in structure-aided drug design, so it was paramount that a robust crystal system was developed to support the diverse hit-finding and iterative optimization process. Increasingly, antibody fragments have been shown to facilitate the crystallization of difficult targets (Kovari *et al.*, 1995 ▸; Rasmussen *et al.*, 2007 ▸; Ring *et al.*, 2013 ▸), so it was decided to pan a phage-display antibody library to find scFv and Fab fragments that might promote a more usable crystal form.

Here, we report the characterization of an antibody fragment produced *in vitro* against Mcl-1 which was formatted as an scFv and a Fab. Both were co-crystallized with Mcl-1, enabling ligand-independent crystallization. In addition to the system described by Clifton *et al.* (2015 ▸), we describe the construct-design choices that led to successful crystallization and how these were used to support the discovery of AZD5991, which is currently in clinical trials, and discuss our findings in antibody-assisted crystallization more generally.

## 2. Materials and methods   

### 2.1. Expression and purification of proteins   

A chimeric Mcl-1 construct (Supplementary Fig. S1) was produced as described previously (Czabotar *et al.*, 2007 ▸) and will be referred to as Mcl-1 throughout. Fully human Mcl-1 (hmMcl-1) and orthologues from monkey (mkMcl-1), dog (doMcl-1), guinea pig (gpMcl-1), rat (raMcl-1) and mouse (moMcl-1) were expressed in transformed *Escherichia coli* cells and purified using a Glutathione Sepharose column (GE Healthcare) followed by Superdex 75 gel filtration (ÄKTA pure, GE Healthcare). The C-terminally tagged scFv and FAb were expressed in Chinese hamster ovary (CHO) mammalian cells (Abbott *et al.*, 2015 ▸), while the N-terminally tagged scFv was expressed in *E. coli* using a standard PET vector. All were purified using Ni–NTA resin (Qiagen) followed by Superdex 75 gel filtration (ÄKTA pure, GE Healthcare).

The identification and production of the scFvs and Fab have been described elsewhere (Tron *et al.*, 2018 ▸).

### 2.2. Complex formation, crystallization and structure determination   

Crystallization of the noncomplexed antibody fragments CHO-scFv, CHO-Fab and *E. coli*-scFv was achieved using sitting-drop vapour diffusion. The scFvs were stored in 100 m*M* NaCl, 10 m*M* Tris pH 7.5, while the Fab was stored in 150 m*M* NaCl, 20 m*M* Tris pH 7.6 (at a concentration of ∼350 µ*M*). Crystallization drops were set up using a Mosquito robot (TTP Labtech) by mixing 200 nl protein complex with 200 nl well solution from a bespoke sparse-matrix screen (as described in the supporting information to Kirkwood *et al.*, 2015 ▸).

Co-crystallization of the Mcl-1–scFv complex was achieved by hanging-drop vapour diffusion. The Mcl-1–scFv complex was produced by mixing solutions of Mcl-1 (1000 µ*M* in 50 m*M* Tris–HCl, 150 m*M* NaCl, 1 m*M* EDTA pH 8) and the scFv (370 µ*M* in 20 m*M* Tris, 150 m*M* NaCl pH 7.6) in a molar ratio of 1:1.1, giving a slight excess of the scFv. Initial crystallization conditions were found using a Mosquito robot and a sparse-matrix screen (as above).

Crystallization and optimization of the Mcl-1–Fab complex was achieved by mixing Mcl-1 (1000 µ*M*) with the Fab (230 µ*M*) in the same way as for the scFv complex.

Optimized crystallization conditions are shown in Table 1 ▸. The antibody complexes crystallized best using a drop ratio of 100:300 nl (protein:precipitant). All of the crystals described here were cryoprotected using a solution comprising 23%(*v*/*v*) 2,3-butanediol and 77%(*v*/*v*) well solution and were flash-cooled by plunging them into liquid nitrogen. Data reduction and scaling was accomplished using *autoPROC* (Vonrhein *et al.*, 2011 ▸), *XDS* (Kabsch, 2010 ▸), *SCALA*/*AIMLESS* (Evans & Murshudov, 2013 ▸) and *STARANISO* (Tickle *et al.*, 2018 ▸). All phasing was achieved using *Phaser* (McCoy *et al.*, 2007 ▸), while model building and refinement were accomplished using *Coot* (Emsley *et al.*, 2010 ▸) and *REFMAC* (Murshudov *et al.*, 2011 ▸) or *BUSTER* (Bricogne *et al.*, 2017 ▸). Surface complementarity was calculated using *SC* (Lawrence & Colman, 1993 ▸) and ligand restraints were generated using *grade* (Smart *et al.*, 2011 ▸)

*In situ* peptidase treatment was accomplished by adding carboxypeptidase Y (Sigma) in a 1:80 molar ratio. After incubating for 10 min on ice, crystallization trials were set up as described previously.

Introduction of the ligand {compound **1**; 3-[3-(1,2,3,4-tetrahydronaphthalen-1-yloxy)propyl]-7-(1,3,5-trimethyl-1*H*-pyrazol-4-yl)-1*H*-indole-2-carboxylic acid} was accomplished using a soaking technique by transferring crystals of the Mcl-1–scFv complex into a soaking solution comprising 15% PEG 2000 MME, 100 m*M* PCTP (sodium propionate, sodium cacodylate trihydrate, bis-Tris propane) buffer pH 6.0 supplemented with 2.5 m*M* compound **1** (stock solution at 100 m*M* in DMSO). The crystals were soaked overnight at 293 K. Data-collection details and statistics can be found in Table 1 ▸.

### 2.3. Surface plasmon resonance (SPR)   

A Biacore 8K instrument (GE Healthcare) was used to monitor binding interactions using a direct binding-assay format. His_6_-tagged Mcl-1 protein (or orthologues) was immobilized using NTA capture-coupling at a flow rate of 10 µl min^−1^ and using an immobilization running buffer consisting of 10 m*M* HEPES, 300 m*M* NaCl, 1 m*M* TCEP, 0.05%(*w*/*v*) Tween 20 at 25°C. Briefly, the sensor surface was activated with a 1 min injection of 0.5 m*M* NiCl_2_ and a 7 min injection of a mixture of 11.5 mg ml^−1^ *N*-hydroxysuccinimide and 75 mg ml^−1^ 1-ethyl-3-(3-dimethylaminopropyl)carbo­diimide hydrochloride. Approximately 300 response units of Mcl-1 protein (2 µg ml^−1^ in immobilization running buffer) were immobilized using the ‘aim for’ function in the *8K Control Software* (GE Healthcare). Remaining reactive esters were blocked using a 7 min injection of 1 *M* ethanolamine. Reference flow cells were prepared without protein. All binding measurements were performed in 10 m*M* Tris pH 7.5, 300 m*M* NaCl, 1 m*M* TCEP, 1% DMSO, 0.02%(*v*/*v*) Tween 20 at 25°C at a flow rate of 30 µl min^−1^. Buffer was primed through the instrument overnight to stabilize the surface before the subsequent assay steps. Prior to kinetic analysis, solvent-calibration and double-referencing subtractions were made to eliminate bulk refractive-index changes, injection noise and data drift. Affinity and binding kinetic parameters were determined by global fitting to a steady-state model within the *Biacore 8K Evaluation Software* (GE Healthcare). The same methods were used for all of the other orthologues.

### 2.4. Isothermal titration calorimetry (ITC)   

ITC was performed using a MicroCal iTC200. The sample cell contained His_6_-tagged Mcl-1 at 15.5 µ*M* and the scFv was titrated 2 µl at a time from a stock at 307 µ*M*. The reaction was performed in 10 m*M* Tris pH 7.4, 100 m*M* NaCl. Data were fitted using *OriginLab.*

### 2.5. Differential scanning fluorimetry (DSF)   

DSF was performed using a Roche Light Cycler 480. The fluorescence excitation and emission filters were set for SYPRO Orange (Sigma). 384-well Framestar PCR microplates (4titude) were used with 10 µl reactions per well. The plates were centrifuged at 180*g* for 1 min before being sealed and used. Each reaction contained protein at 10 µ*M* and SYPRO Orange at 10× in 100 m*M* NaCl, 10 m*M* Tris pH 7.5 unless otherwise stated. The temperature was increased from 20 to 90°C at a rate of 1.5°C min^−1^. *Genedata Screener* (Genedata) was used to evaluate the data, in which a melting temperature (*T*_m_) for each well is determined by calculating the first-derivative function of the melting trace. For single-transition melting curves this describes a peak which defines the *T*_m_ of each condition.

### 2.6. Analytical size-exclusion chromatography of the Fab and the scFv   

In order to further refine the crystallization of the Mcl-1–scFv and Mcl-1–Fab complexes, analytical size-exclusion chromatography was performed to better determine the ratio of the components. All previous crystallizations had relied on mixing molar ratios of Mcl-1 and the Fab or scFv. However, OD_280_ measurements are prone to error owing to the presence of inactive protein or other contaminants. It would have been possible to perform a larger scale size-exclusion experiment and capture the eluent corresponding to the complex. However, preliminary tests suggested that the Mcl-1–Fab complex did not recover well after being stored frozen, so it was decided to continue mixing the components but in an empirically determined ratio.

**Figure 1:**
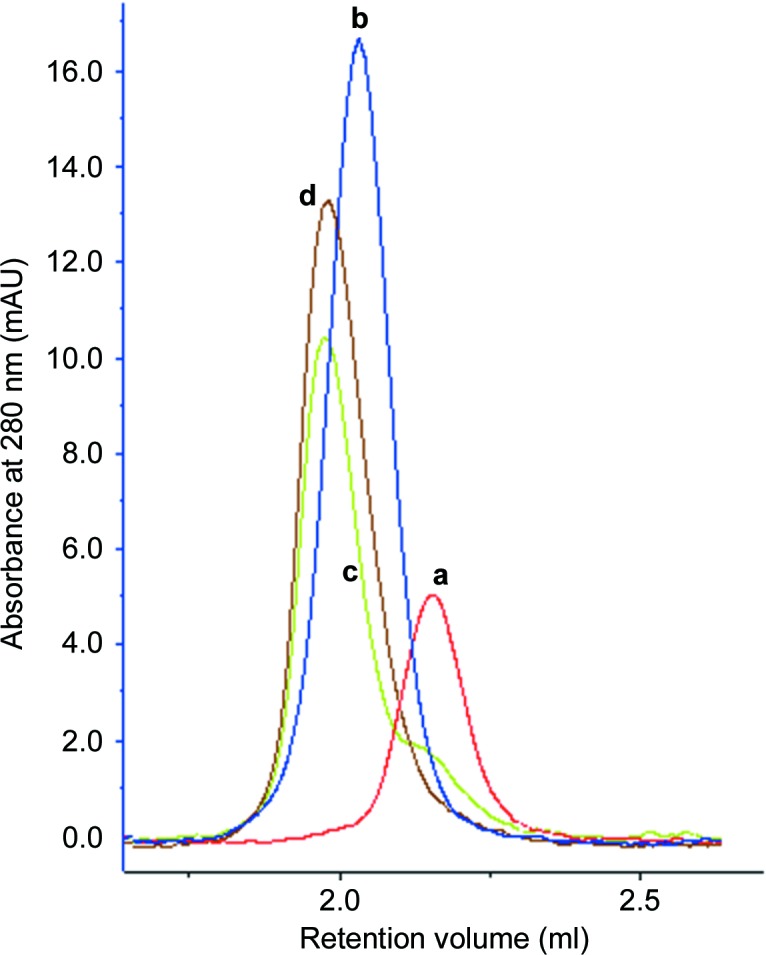
Chromatogram showing size-exclusion traces for the Mcl-1–Fab complex and controls. Peaks **a** (red) and **b** (blue) correspond to Mcl-1 and Fab, respectively. Volumetric Mcl-1:Fab ratios were 1:4 (peak **d**, brown) and 1:3 (peak **c**, green). The shoulder on peak **c** is indicative of residual Mcl-1 compared with peak **d**, which suggests a fully reacted mixture.

In order to determine the ratio of active components, varying volumetric ratios of Mcl-1 (1000 µ*M*) and Fab (230 µ*M*) were prepared and 20 µl samples were run at 1 ml min^−1^ on a 5 × 150 mm S200 column (ÄKTAexplorer, GE Healthcare). The running buffer was 10 m*M* Tris–HCl, 150 m*M* NaCl pH 7.8 and the absorbance was measured at 280 nm. An example of the results for the Mcl-1–Fab SEC are shown in Fig. 1 ▸. Using data from the size-exclusion experiments, it was possible to refine the molar mixing ratios from 1:1.1 (equivalent to a volumetric ratio of 1:3.7) to a new volumetric ratio of 1:4, which had a positive impact on the crystallization. Analytical size exclusion was also used to calculate refined mixing ratios when new batches of the proteins were produced during the campaign.

## 3. Results   

### 3.1. Biophysical characterization of the scFv and Mcl-1   

The scFv was discovered using phage display coupled with size-exclusion chromatography (SEC) and the *K*_d_ for its interaction with Mcl-1 was not known.

**Figure 2:**
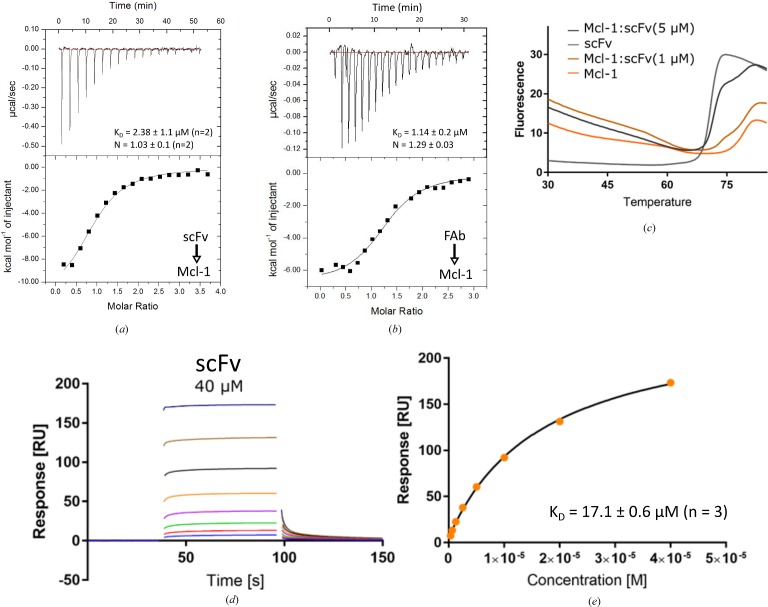
Biophysical characterization of the binding of the scFv to Mcl-1. Isothermal plots for the titration of the scFv (*a*) and Fab (*b*) into a solution of Mcl-1. (*c*) Thermal shift assay fluorescence plot versus temperature. (*d*) Binding sensorgrams of scFv binding to immobilized Mcl-1. Representative traces are shown (see Supplementary Figs. S2 and S3). The top concentration tested was 40 µ*M* with a twofold dilution. (*e*) Steady-state fit of scFv binding to Mcl-1.

Isothermal titration calorimetry (ITC) and surface plasmon resonance (SPR) were used to evaluate the binding of the scFv to Mcl-1, huMcl-1 and Mcl-1 orthologues. All of the ortho­logues tested in SPR showed a similar *K*_d_ for the scFv. The addition of saturating concentrations of AZD5991 in an A–B–A experiment did not show significant changes in the *K*_d_ of the scFv towards any of the orthologues. ITC was used to determine the *K*_d_ of the Fab with Mcl-1. Isotherms from the ITC experiment showed the scFv and Fab to be relatively weak binders, with *K*_d_ values of around 2.38 ± 1.1 and 1.14 ± 0.2 µ*M*, respectively (Figs. 2 ▸ *a* and 2 ▸ *b*). In SPR for the scFv (Figs. 2 ▸ *d* and 2 ▸ *e*), plotting the steady-state responses resulted in a best fit for *K*_d_ = 17.1 ± 0.6 µ*M*. Mcl-1, the scFv and their complex were tested using differential scanning fluorimetry. The *T*_m_ values for the scFv and Mcl-1 were determined to be 70.5°C (SD = 0.01; *n* = 3) and 78.2°C (SD = 0.01; *n* = 3), respectively. Interestingly, the complex showed a more complex double transition, suggesting sequential unfolding of both components. In an excess concentration of Mcl-1, the transition corresponding to scFv shifted from 70.5 to 73.8°C (Fig. 2 ▸ *c*). Details of the biophysical data can be found in Supplementary Figs. S2 and S3.

### 3.2. The apo structure of Mcl-1 bound to the scFv   

**Figure 3:**
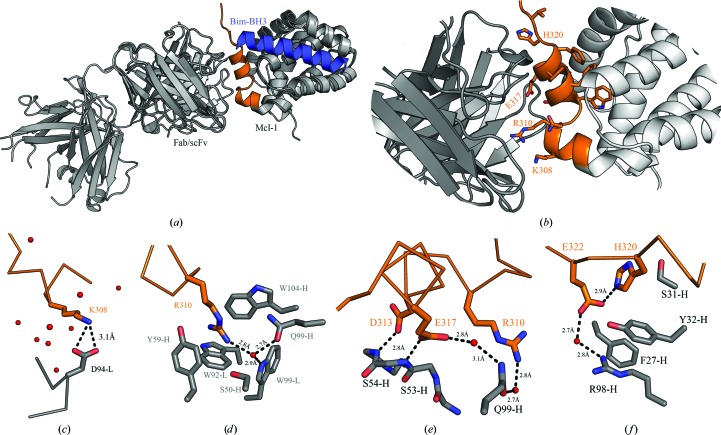
The structures of the Mcl-1–scFv and Mcl-1–Fab complexes. Mcl-1 is bound by the scFv (PDB entry 6qb3) or Fab (PDB entry 6qb6) via the BH2 helix. (*a*) Models of Mcl-1–scFv and Mcl-1–Fab overlaid. The epitope in the C-terminal region is shown in orange. The V_H_ and V_L_ regions of the Fab are structurally very similar to those in the scFv, suggesting that the addition of the constant domains had little effect on the binding mode. For reference, a natural Bim BH3 peptide ligand (blue) is overlaid (PDB entry 2pqk; Fire *et al.*, 2010 ▸). (*b*) Residues from the epitope interaction are annotated and shown in orange stick representation. Arg310 is in an extended conformation and is buried in the scFv CDR binding site. (*c*–*f*) Stick representations of the main interactions between Mcl-1 (orange) and scFv/Fab (grey). Water atoms are shown as red spheres.

The apo structure of the Mcl-1–scFv complex (PDB entry 6qb3) was crystallized from 10–15%(*w*/*v*) PEG 200 MME, 0.1 *M* PCTP buffer pH 5–6 and was solved by molecular replacement using starting models derived from PDB entry 2nl9 (Czabotar *et al.*, 2007 ▸) for Mcl-1 and PDB entry 4uu9 (Colley *et al.*, 2018 ▸) for the scFv. The crystal structure revealed a single scFv molecule bound to Mcl-1, with a single complex in the asymmetric unit (Fig. 3 ▸ *a*).

Overall, the structure showed good electron density and it was possible to model the majority of the complex. Missing density was observed for the N- and C-termini and the Gly-Ser linker of the scFv. In common with the previous structures of Mcl-1, density for Ser196–Gly203 was also missing in the refined structure. These residues are part of a flexible loop between helices 1 and 2 (residues 196–204) that contains the mouse–human substitutions.

The epitope–paratope interaction between the scFv and Mcl-1 were seen to be almost entirely derived from helix 8, the BH2 helix, of Mcl-1, with a shape-complementarity statistic Sc of 0.644 (Lawrence & Colman, 1993 ▸; Fig. 3 ▸ *b*).

To make comparison between the scFv and Fab easier, all of the following descriptions of the scFv are annotated using the Fab heavy-chain (H) and light-chain (L) amino-acid numbering. A sequence alignment with numbering for the Fab and scFv can be found in Supplementary Fig. S1.

One striking feature of the Mcl-1–scFv interaction is the extended side chain of Arg310 of Mcl-1, which is buried in a deep pocket made up from three scFv tryptophan residues: Trp92-L, Trp99-L and Trp104-H (Fig. 3 ▸ *d* and Supplementary Fig. S4). The guanidinium head group is coordinated via a network of water molecules and Gln99-H and Trp99-L. Comparing the conformation of Arg310 of Mcl-1 in the scFv structure with all other Mcl-1 PDB depositions shows a wide variation of side-chain conformations beyond the C^γ^ atom, suggesting that Arg310 is mobile until captured by the epitope interaction. Another key interaction is that of Lys308 of Mcl-1 with Asp94-L and Asn167-L of the scFv (Asn167-H is not shown for clarity), which form a salt bridge (Fig. 3 ▸ *c*). A pair of acidic residues, Asp313 and Glu317, in Mcl-1 form an extended network of hydrogen bonds though coordinated waters with Gln99-H of the scFv, Arg310 of Mcl-1 and the backbone of the CDR regions of the scFv. A further intramolecular interaction between His320 and Glu322 of Mcl-1 extends via a water bridge to Arg98-H in the scFv.

Interestingly, the intramolecular interactions coordinated by Asp313, Glu317 and His320 of Mcl-1 are involved in the coordination of the crystallization-derived zinc ion seen in the Mcl-1–BH3 inhibitor-peptide structures with PDB codes 3kj1, 3io9, 2pqk and 2nl9 (Fire *et al.*, 2010 ▸; Lee *et al.*, 2009 ▸; Czabotar *et al.*, 2007 ▸).

### 3.3. The antibody-enabled Mcl-1 crystal system supports the soaking of compounds   

**Figure 4:**
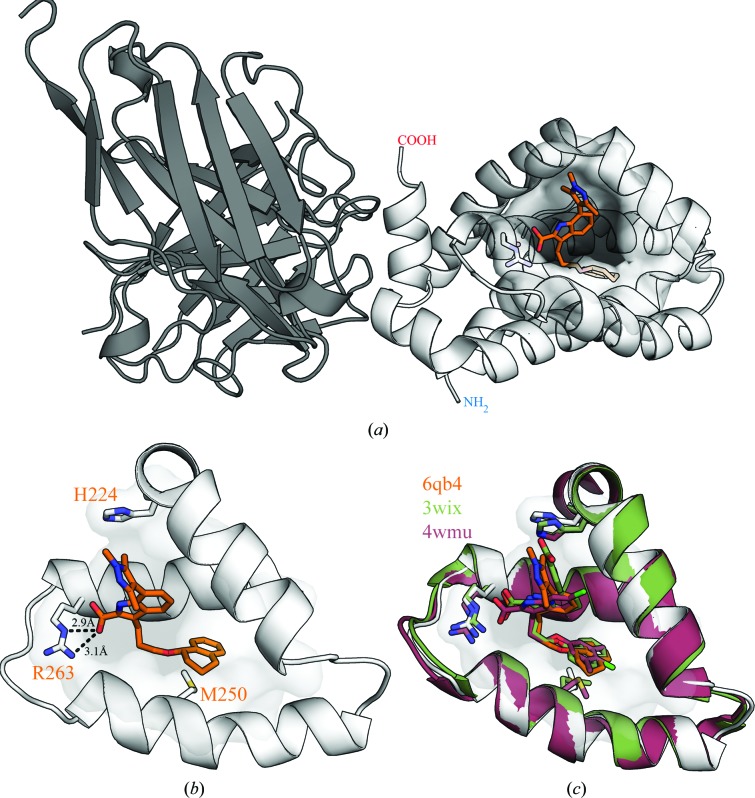
The soaked structure of an indole acid inhibitor binding to the Mcl-1–scFv complex (PDB entry 6qb4). (*a*) Ribbon representation of the whole complex, with Mcl-1 (light grey) bound by the scFv (dark grey) and the inhibitor (orange). A surface representation is displayed around the ligand-binding site. (*b*) Detailed view of the inhibitor in the BH3 binding site, showing the critical salt-bridge interaction between the indole acid group and Arg263 and the deep hydrophobic pocket that accommodates the tetrahydronaphthyl anchor. (*c*) Overlay showing the structural similarity between the Mcl-1–scFv complex and published structures with analogous inhibitors. Green, PDB entry 3wix co-crystallized with Mcl-1 (Tanaka *et al.*, 2013 ▸); dark red, PDB entry 4wmu co-crystallized using an Mcl-1-MBP fusion system (Clifton *et al.*, 2015 ▸); orange, Mcl-1–scFv crystals soaked with inhibitor.

To determine the suitability of the antibody-enabled Mcl-1 crystal system for iterative structural support in drug discovery, a simple soaking experiment was performed. Apo crystals of the Mcl-1–scFv complex were soaked overnight in a solution containing 2.5 m*M* of an indole acid inhibitor similar to those reported in the literature. The ligand was chosen as an exemplar of the indole acid-type compounds in the literature at the time (2012). The pyrazole substitution on the indole was a key vector for the design around AZD5991. The resulting crystal structure was solved at 2.38 Å resolution and is shown in Fig. 4 ▸(*a*) (PDB entry 6qb4). The binding mode closely matched those of previously reported indole acid inhibitors; the indole acid makes a salt-bridge interaction with Arg263 and the tetrahydronaphthalene hydrophobic anchor is buried deep in the hydrophobic core of the BH3 binding site (Figs. 4 ▸ *b* and 4 ▸ *c*). A simple OMIT map of the ligand-binding site can be seen in Supplementary Fig. S5.

### 3.4. A second apo structure revealed the C-terminal tag remnant occupying the active site   

**Figure 5:**
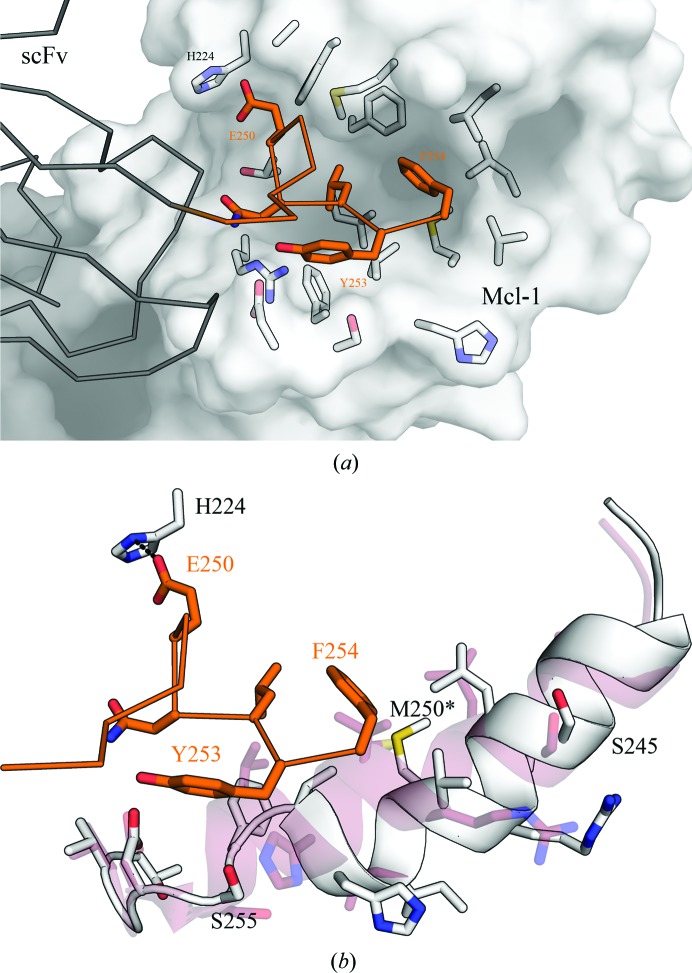
An overlay showing the displacement of the Mcl-1 BH3 helix by a symmetry-related copy of the C-terminal scFv tag remnant. (*a*) Overview of the crystal contact between a symmetry-related molecule, shown as a C^α^ trace, and the BH3 binding pocket of Mcl-1, shown as a surface. By chance, the tag remnant is positioned such that it is able to make an ordered interaction with the pocket. (*b*) A more detailed view of the interaction between the scFv tag remnant and the BH3 pocket of Mcl-1. The tag remnant is shown as an orange C^α^ trace with side chains as sticks, and the BH3 binding site of Mcl-1 is shown as a white cartoon with side chains as sticks. The apo Mcl-1–scFv complex (PDB entry 6qb3) is overlaid in rose, showing the binding effect on the BH3 binding groove. Although there is a considerable displacement and conformational rearrangement of the helix, the S^δ^ atom of Met250 remains in approximately the same place (highlighted with an asterisk), demonstrating the plasticity of the ligand-binding pocket. The interactions are mostly hydrophobic, but a hydrogen bond (2.8 Å) was observed between His224 of Mcl-1 and Glu250 of the scFv (dashed line).

Surprisingly, a second apo structure determined at 1.96 Å resolution from the same materials showed an intriguing feature. The TEV cleavage-site remnant G_246_AAAENLYF_254_ from a symmetry-related molecule had bound in the Mcl-1 BH3 binding site (Fig. 5 ▸). The amino acids were clearly ordered, allowing unequivocal fitting of the sequence. The electron density of the earlier apo Mcl-1–scFv structure (PDB entry 6qb3) showed that the interpretable density terminated at Leu245.

Although an artefact of the crystallization, this observation suggests how malleable the BH3 binding site is. In accommodating the short length of polypeptide, Leu252, Tyr253 and Phe254 displace and partially melt helix 4 (Ser245–Ser255). The effect is to widen the pocket by displacing Ile251, His252 and Val253 outwards by around 3 Å and allowing the position of the Met250 C^α^ atom to move by around 3.6 Å. However, the position of the S^δ^ atom is unchanged, which appears to keep the hydrophobic packing in this region intact, accommodating Leu252 and Phe254. Finally, an electrostatic interaction was observed between Glu250-L of the Fab and His224 of Mcl-1. A simple OMIT map of this region and an overlay showing the C^α^ traces of the BimBH3 binding mode (PDB entry 2nl9) and the C-terminal scFv tag (PDB entry 6qfc) can be found in Supplementary Figs. S6 and S7.

Although it had been demonstrated that it was possible to generate ligand-bound structures using the scFv, even by soaking, the presence of the TEV cleavage remnant was a concern for the project. Attempts to crystallize the scFv material with the full C-terminal His_6_ tag were unsuccessful, so the expression of an N-terminally His_6_-tagged scFv in *E. coli* was tested. The *E. coli*-expressed material was tested both tagged and detagged in crystallization using the same protocol as described earlier, both in the presence and absence of ligands. However, after considerable efforts in crystallization screening no crystals of the complex were obtained.

### 3.5. Conversion of the scFv to a Fab   

**Figure 6:**
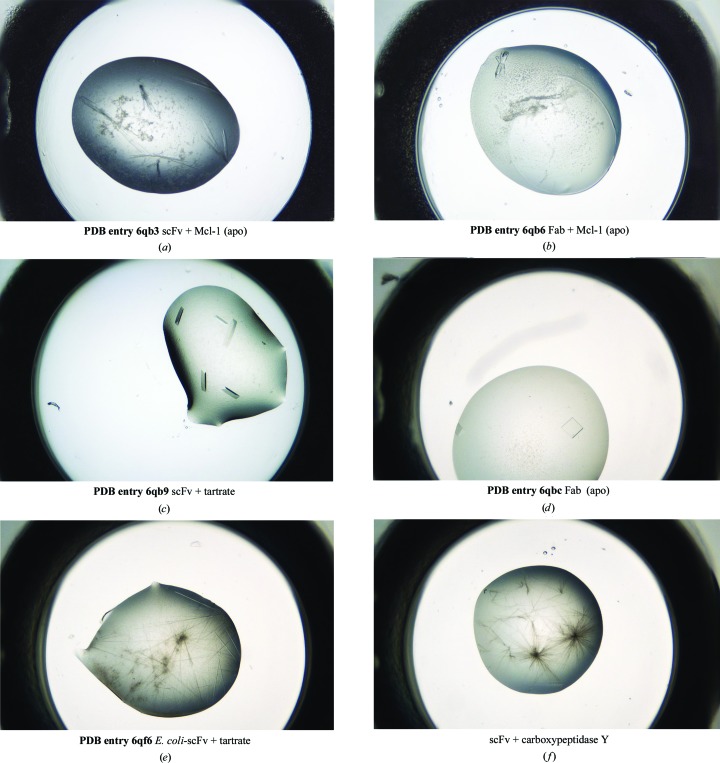
Photomicrographs showing the crystal morphologies of the samples used in structure determination and their crystallographic properties. In each case the crystallization conditions, space group, unit-cell parameters and resolution are given. (*a*) PDB entry 6qb3; scFv in complex with Mcl-1, apo; 10–15% PEG 200 MME, 0.1 *M* PCTP buffer pH 5–6; *C*2; *a* = 143.08, *b* = 40.38, *c* = 75.42 Å, α = γ = 90, β = 110.50°; resolution 1.90 Å. (*b*) PDB entry 6qb6; Fab in complex with Mcl-1, apo; 10–15% PEG 200 MME, 0.1 *M* PCTP buffer pH 5–6; *C*2; *a* = 148.05, *b* = 42.46, *c* = 106.23 Å, α = γ = 90, β = 113.19°; resolution 2.24 Å. (*c*) PDB entry 6qb9; scFv with bound tartrate; 1 *M* potassium/sodium tartrate, 0.1 *M* HEPES pH 7.5; *C*2; *a* = 77.88, *b* = 63.03, *c* = 101.70 Å, α = γ = 90, β = 109.96°; resolution 1.85 Å. (*d*) PDB entry 6qbc; Fab structure; 15%(*w*/*v*) PEG 3350, 0.1 *M* MgCl_2_, 0.1 *M* PCPT buffer pH 7.5; *P*3_1_21; *a* = *b* = 70.54, *c* = 168.34 Å, α = β = 90, γ = 120°; resolution 1.56 Å. (*e*) PDB entry 6qf6; *E. coli*-scFv; 0.4 *M* ammonium sulfate, 25%(*w*/*v*) PEG 3350, 0.1 *M* bis-Tris pH 5.9; *P*4_3_2_1_2; *a* = *b* = 180.18, *c* = 88.42 Å, α = β = γ = 90°; resolution 2.59 Å. (*f*) CHO-expressed scFv after treatment with carboxypeptidase Y; 0.2 *M* lithium chloride, 25% PEG 3350, 0.1 *M* bis-Tris pH 6.5.

Standard molecular-biology techniques were used to add mouse constant domains to the scFv V_H_ and V_L_ domains, yielding a Fab. The heavy and light chains were co-expressed using a CHO cell line and purified. After cleaving the light-chain C-terminal His_6_ tag, the Fab was crystallized with Mcl-1 by mixing stoichiometric ratios and screening for crystallization conditions as described previously. Interestingly, the only successful condition identified for the apo Mcl-1–Fab complex was identical to the condition used to crystallize the scFv complex, namely PEG 2000 MME, 100 m*M* PCTP buffer pH 5–6 (Fig. 6 ▸ *b*). *In situ* diffraction screening showed diffraction to around 6 Å resolution using a 30 s exposure on an in-house source (data not shown; Hargreaves, 2012 ▸). Crystals from the same crystallization drop were cooled after cryoprotection using 23% butane-2,3-diol and sent for data collection at the Diamond Light Source (DLS) synchrotron. The crystal structure showed that the crystals belonged to the same space group as the scFv complex, with similar unit-cell parameters apart from an increase in *c* from 75.4 to 106.2 Å (see Table 1 ▸ and Fig. 6 ▸). Comparison of the scFv and Fab complexes showed no significant differences in the positions of the V_H_ and V_L_ domains (with an r.m.s.d. on C^α^ atoms of 0.41 Å). However, Mcl-1 bound to the Fab complex showed a more complete chain trace, which included the Mcl-1 residues Ser196–Ala204 that were not seen in the structure of the scFv complex. These elements appear to be stabilized by crystal-packing inter­actions in the Fab complex crystal. Comparison of the Mcl-1–Fab complex structure with the apo MBP fusion-protein structure (PDB entry 4wms; Clifton *et al.*, 2015 ▸) shows a similar chain trace for the Ser196–Ala204 region.

### 3.6. Structures of the unbound mammalian-expressed scFv and Fab   

As a control experiment, all of the individual components of the complexes were also screened for crystallization separately. Mammalian-expressed CHO-scFv and CHO-Fab and *E. coli*-expressed *E. coli*-scFv were screened both tagged and detagged. Crystal structures were determined for detagged CHO-scFv (1.35 Å resolution; PDB entry 6qb9), tagged CHO-Fab (1.56 Å resolution; PDB entry 6qbc) and detagged *E. coli*-scFv (2.59 Å resolution; PDB entry 6qf6).

**Figure 7:**
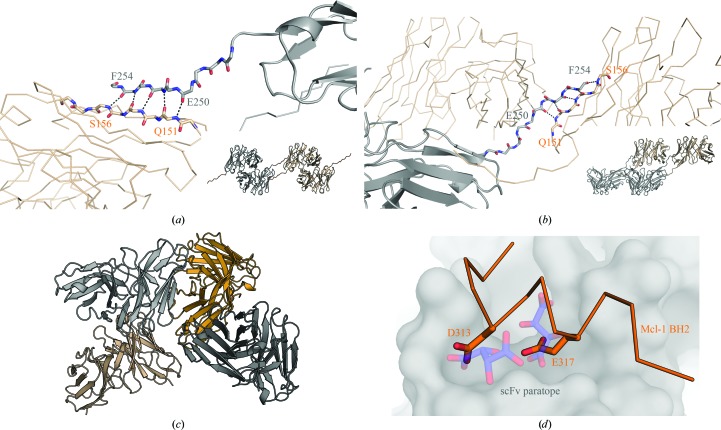
The C-terminal tag-cleavage remnants drive multiple crystal forms though β-extension packing in crystal contacts. (*a*) Ribbon and stick representation showing the C-terminal tag remnants forming a β-sheet extension with a symmetry-related molecule in the *P*2_1_ crystal form (PDB entry 6qf9) grown from PEG 4000 (resolution 1.4 Å). The inset shows the overall packing. (*b*) Ribbon and stick representation showing the *C*2 crystal form of the scFv (PDB entry 6qb9; resolution 1.85 Å) grown from tartrate. The inset shows the overall packing. (*c*) N-terminally tagged and cleaved *E. coli*-scFv crystal structure in space group *P*4_3_2_1_2 showing four monomers in the asymmetric unit (PDB entry 6qf6; resolution 2.6 Å). (*d*) Surface representation of the scFv CDR region of PDB entry 6qb9 with the two tartrate molecules from the crystallization solution shown as sticks. Overlaid in orange is the Mcl-1 epitope from the complex structure shown as a C^α^ trace. Asp313 and Glu317 normally occupy the tartrate-binding sites in the complex (shown as side-chain sticks).

CHO-scFv initially crystallized in 1 *M* potassium/sodium tartrate, 0.1 *M* HEPES pH 7.5 (Fig. 6 ▸ *c*). The crystals belonged to space group *C*2, with two monomers in the asymmetric unit (Fig. 7 ▸ *b*). Intriguingly, two tartrate ions derived from the crystallization conditions (average *B* factor 23.8 Å^2^) occupied the space occupied by the Mcl-1 acidic side chains Asp313 and Glu317 in the antibody-complex structure (Fig. 7 ▸ *d*), appearing to mimic the Mcl-1 acidic side-chain interactions.

CHO-scFv was also crystallized in 25%(*v*/*v*) PEG 4000, 0.1 *M* HEPES pH 7.5, 10%(*v*/*v*) 2-propanol. The crystals were cryoprotected as described above and data were collected. The structure was solved in space group *P*2_1_, with two monomers in the asymmetric unit (PDB entry 6qf9). Unlike the tartrate-bound crystals (PDB entry 6qb9), the binding CDRs in this crystal form were exposed and free for any potential ligand to bind. Crystals of this form were soaked with 20 m*M* sodium aspartate, 20 m*M* sodium glutamate and 20 m*M* arginine. The resulting structures did not show any specific binding of glutamine, asparagine or arginine. However, a higher concentration of arginine (50 m*M*) was used in co-crystallization, which did appear to show weak density for the guanidinium head group only. The refinement, however, was not sufficiently convincing for public deposition (data not shown).

To further investigate the paratope–epitope interaction observed in the Mcl-1 antibody complexes, two peptides were designed based on the Mcl-1 BH2 epitope sequence. The first was a direct copy of the BH2 helix and the second was a shortened and less hydrophobic version in which the bulky tryptophan and phenylalanine were replaced with alanines (peptide 1, VKQRGWDGFVEFFHVE; peptide 2, RGADGAVEA). No binding was observed in the electron-density maps from co-crystallization with the scFv, and subsequent biophysical analysis using SPR and ITC also showed no measurable binding.

However, when the CHO-Fab was screened in co-crystallization trials with peptide 1, crystals appeared that had not been obtained without the peptide. Crystals grown in the presence of peptide 1 (1 m*M*) from 15%(*w*/*v*) PEG 3350, 0.1 *M* MgCl_2_, 0.1 *M* PCPT buffer pH 7.5 (Fig. 6 ▸ *d*) were cryoprotected as described previously and data were collected to 1.56 Å resolution. The crystals belonged to space group *P*3_1_21, with a monomer in the asymmetric unit (PDB entry 6qbc). The Fab V_H_/V_L_ chains were structurally very similar to those in the CHO-scFv, giving a C^α^ r.m.s.d. of 0.41 Å. However, no density was observed for the peptide. The CHO-Fab had been screened for crystallization many times over the course of the project and only produced crystals on this occasion. It was therefore assumed that the peptide had some influence on the crystallization, perhaps promoting nucleation.

### 3.7. The importance of the C-terminal tag remnant and the *E. coli*-scFv crystal structure   

The construct expressed using the *E. coli* PET vector was N-terminally His_6_-TEV-tagged, while the CHO-scFv was C-terminally His_6_-TEV-tagged. C-terminally tagged constructs incorporating a TEV cleavage site leave five residues of the TEV recognition sequence post-cleavage, whereas N-terminally tagged and cleaved constructs leave only GS at the N-terminus. Interestingly, the C-terminal tag remnant formed extensive crystal contacts in both the *C*2 and *P*2_1_ CHO-scFv crystal forms, as well as being crucial to crystallizing the scFv complex. It is worth noting that the original scFv panning used a His_6_-TEV C-terminal tag, while the N-terminus has a leader sequence that flags it for periplasmic export. The C-terminal tag remnant G_246_AAAENLYF_254_ was observed to interact with the BH3 binding site in the Mcl-1–scFv complex. In the scFv structures the tag remnant made a crystal contact with a symmetry-related molecule via β-sheet extension with residues Q_151_RVTIS_156_ (Figs. 7 ▸ *a* and 7 ▸ *b*). The importance of this contact in driving crystallization became apparent with the crystal structure of the N-terminally His_6_-tagged and cleaved *E. coli*-scFv. This construct only crystallized as fine fragile needles (Fig. 6 ▸ *e*) grown from 0.4 *M* ammonium sulfate, 25% PEG 3350, 0.1 *M* bis-Tris pH 5.9. Multiple samples were cryoprotected in butane-2,3-diol, flash-cooled and screened for diffraction at DLS. Eventually, a suitable crystal was identified for data collection using a helical scan, and a data set to a resolution of 2.69 Å was obtained. The crystals belonged to space group *P*4_3_2_1_2, with four monomers in the asymmetric unit (Fig. 7 ▸ *c*). It was clear from the structure that the crystal packing could not accommodate a C-terminal tag remnant and that the tag remnant was critical in enabling the higher resolution crystal from. It was hypothesized that removing the C-terminus from the CHO-scFv would result in the production of the needle-like crystal form. When the CHO-scFv was treated with carboxypeptidase Y during crystallization trials, only crystals with a needle-like morphology appeared (Fig. 6 ▸ *f*), suggesting that the peptidase had worked and the loss of the C-terminal tag remnant had resulted in the needle-like crystal morphology. The CHO-scFv crystals generated using carboxy­peptidase Y were too small and fragile to manipulate and it was not possible to obtain a suitable sample for data collection.

## 4. Discussion   

Mcl-1 is an important oncology drug target but is only crystallizable with peptides or small molecules, as a fusion protein or, as described here, with antibody fragments. In recent years there has been increasing interest in using antibody fragments to enable the crystallization of structurally intractable targets (Griffin & Lawson, 2011 ▸). Over the last eight years Astra­Zeneca has made considerable efforts to facilitate a number of drug-target crystal systems using antibodies. Although all of the diverse targets pursued have resulted in scFvs or Fabs that appear to have the correct properties for crystallization, most have failed to produce crystals. The low success rate is most likely to be a product of the technique being used as a rescue strategy when all other attempts at crystallization had failed. In this light, a low success rate is an acceptable consequence.

In this paper, we have demonstrated the considerable effect that the tagging protocol can have on crystallization and how the conversion of an scFv to a full Fab can have positive effects. However, the opposite can also be true. In previous work (Colley *et al.*, 2018 ▸), the highly potent and specific Fab for cytokine C5a would not crystallize even though a well characterized and highly purified sample was produced. In that case the conversion of the Fab to an scFv resulted in well diffracting crystals. In the case of Mcl-1 reported here, both scFvs and Fabs, as well as an apo Mcl-1 crystal system, were useful in supporting different hit-identification strategies. Although the Fab system was the most useful in supporting the lead-optimization phase, resulting in the macrocyclic clinical candidate, the apo Mcl-1 and the scFv system were critical earlier in the project, supporting both HTS and DNA-encoded library output with a total of over 80 ligand-bound structures.

It is interesting to consider what contributes to the success of the Mcl-1 antibody system. Firstly, and perhaps most importantly, Mcl-1 is a flexible yet robust protein with a *T*_m_ in excess of 70°C. This almost certainly facilitates phage-display panning approaches, which require the protein to remain intact during the selection and washing stages. Furthermore, Mcl-1 is a small compact protein functionally evolved for protein–protein interaction, which may facilitate the selection of three-dimensional epitopes as opposed to linear epitopes derived from flexible loops, unfolded protein or termini. The small size also results in a larger impact when suitable binders are identified. The ratio of the volume between the target and the antibody fragment may well impact on the crystallizability since more of the crystal contacts are likely to be formed through the well behaved antibody fragments rather than the uncrystallizable target. This was indeed the case in the Fab–IL17 structure, in which all of the crystal contacts were mediated via the Fab (Gerhardt *et al.*, 2009 ▸; PDB entry 2vxs).

Part of the antibody-panning process for Mcl-1 was a validation step using size-exclusion chromatography, in which 40 scFvs expressed using microscale periplasmic methods were tested for complex formation (see the supporting information to Johannes *et al.*, 2017 ▸). This resulted in four candidates that showed shifts, and of these four only one produced crystals. This step is resource-intensive, and other surrogate measures of complex formation such as affinity ranking measured by SPR off-rates have been used. Furthermore, there is no surrogate measure of crystallizability, so even if the scFv appears to have good affinity and forms stable complexes they can still frustratingly resist crystallization. However, once a stable complex has been formed antibody fragments can be manipulated quite easily. Adding constant domains to the initial scFv produced a second crystal system and a point mutation (variable light chain) E82A was successfully introduced to remove a symmetry-related acidic residue from the region close to the pyrazole in PDB entry 6qb4 (data not shown). This was performed to fully explore vectors from the pyrazole without being constrained by the glutamate in the Mcl-1–Fab complex system.

Further crystal engineering can be achieved through consideration of the position of the affinity tags as well as detagging. In the crystallization of the individual antibody fragments, the positioning of the tag on the C-terminus resulted in a tag remnant after cleavage, while no remnant is left behind when the tag is on the N-terminus. As shown by both crystal forms of the CHO-scFv this contributed to the crystal packing, and its removal with carboxypeptidase Y led to a less favourable crystal form and no crystals at all in complex with Mcl-1. In the case of the Mcl-1–Fab system it was not necessary to cleave the light-chain C-terminal tag in order to obtain crystals. In fact, cleavage of the tag caused a significant loss of protein owing to precipitation, so the tag was left in place thereafter.

The *E. coli*-scFv construct was unsuccessful in crystallization with Mcl-1. Initially, this was thought to be owing to malformed disulfide bonds; the expression vector used for this construct was a standard PET system rather than the periplasmic export system used for antibody screening (Marco, 2009 ▸). The structure obtained from the *E. coli*-expressed material suggested that disulfide formation was not an issue. However, the PET-expressed material failed to produce complex crystals with Mcl-1 and was difficult to crystallize on its own, which might suggest that the disulfide formation was heterogeneous in the sample.

The tag remnant appears crucial, such that a construct designed for expression in *E. coli* would most likely need the C-terminal tag. Certainly, a PET expression system would be preferable to the periplasmic expression system used on a small scale during the panning process, as the PET system had a better yield in this particular case and is preferable in terms of cost compared with the CHO system. However, switching to the CHO expression system enabled full Fabs to be expressed as well as scFvs, at scale, with correct folding and disulfide formation.

## Supplementary Material

PDB reference: Mcl-1 in complex with an scFv, apo, 6qb3

PDB reference: liganded, 6qb4

PDB reference: Mcl-1 in complex with a Fab, 6qb6

PDB reference: scFv with bound tartrate, 6qb9

PDB reference: Fab, 6qbc

PDB reference: *E. coli*-expressed scFv, 6qf6

PDB reference: scFv, 6qf9

PDB reference: Mcl-1–scFv complex showing C-­terminal tag binding, 6qfc

Supplementary Figures. DOI: 10.1107/S2059798319014116/jb5013sup1.pdf

## Figures and Tables

**Table:** 

PDB code	6qb3	6qb6	6qb4	6qb9
Description	scFv in complex with Mcl-1, apo	Fab in complex with Mcl-1, apo	Compound **1** soaked into scFv–Mcl1 complex	scFv with bound tartrate
Crystallization conditions	10–15% PEG 200 MME, 0.1 *M* PCTP buffer pH 5–6	10–15% PEG 200 MME, 0.1 *M* PCTP buffer pH 5–6	23%(*w*/*v*) PEG 3350, 0.1 *M* MgCl_2_, 0.1 *M* PCPT buffer pH 7.8	1 *M* potassium/sodium tartrate, 0.1 *M* HEPES pH 7.5
Data collection				
Wavelength (Å)	0.92000	0.97900	0.92000	0.97949
Space group	*C*2	*C*2	*C*2	*C*2
*a*, *b*, *c* (Å)	143.08, 40.38, 75.42	148.05, 42.46, 106.23	144.95, 40.86, 77.35	77.88, 63.03, 101.70
α, β, γ (°)	90, 110.50, 90	90, 113.19, 90	90, 111.42, 90	90, 109.96, 90
Resolution range (Å)	38.66–1.90 (1.96–1.90)	50.1–2.24 (2.30–2.24)	42.15–2.38 (2.50–2.38)	38.9–1.85 (1.90–1.85)
No. of reflections	117447	96081	55681	125161
Unique reflections	32161	29461	16889	39035
Multiplicity	2.0 (2.0)	3.3 (3.4)	3.3 (3.4)	3.2 (3.2)
Completeness (%)	99.6	99.3 (99.8)	98.5 (99.4)	98.6 (99.6)
〈*I*/σ(*I*)〉	12.1 (2.4)	12.3 (2.1)	73.0 (2.6)	83.0 (1.7)
*R*_merge_ (%)	8.4 (61.1)	7.6 (60.0)	10.0 (39.0)	9.5 (72.7)
CC_1/2_	N/A	N/A	N/A	0.995 (0.548)
*R*_meas_ (all *I* ^+^ and *I* ^−^)	N/A	N/A	N/A	0.113 (0.870)
Refinement				
Overall Wilson *B* (Å^2^)	21.2	37.0	28.0	25.4
R.m.s. deviations				
Bond lengths (Å)	0.01	0.01	0.01	0.01
Bond angles (°)	0.99	1.14	1.06	1.10
No. of atoms				
Protein	2857	4429	2872	3555
Ligand	0	0	34	20
Water	358	154	157	183
Ligand name [PDB code]	NA	NA	Compound **1** [HVN]	Tartrate [TLA]
*B* factors (Å^2^)				
Protein	39.60	48.50	38.80	30.44
Ligand	NA	NA	44.10	29.77
Water	43.10	41.40	33.00	41.50
*R*_work_/*R*_free_ (%)	17.2/20.5	18.7/23.2	19.7/23.1	20.0/23.9
Ramachandran parameters				
Preferred (%)	97.2	95.6	96.4	95.7
Allowed (%)	2.3	3.5	3.0	2.8

**Table:** 

PDB code	6qbc	6qf6	6qf9	6qfc
Description	Fab structure	*E. coli*-scFv	scFv packing relies on the presence of the C-terminal tag	scFv C-terminal tag binds in the peptide groove
Crystallization conditions	15%(*w*/*v*) PEG 3350, 0.1 *M* MgCl_2_, 0.1 *M* PCPT buffer pH 7.5	0.4 *M* ammonium sulfate, 25%(*w*/*v*) PEG 3350, 0.1 *M* bis-Tris pH 5.9	20%(*w*/*v*) PEG 4000, 10%(*v*/*v*) 2-propanol, 0.1 *M* HEPES pH 7.5	12%(*w*/*v*) PEG 10K, 10%(*v*/*v*) DMSO, 0.1 *M* PCPT buffer pH 7.4
Data collection				
Wavelength (Å)	0.97623	0.97625	0.97950	0.97626
Space group	*P*3_1_21	*P*4_3_2_1_2	*P*2_1_	*C*2
*a*, *b*, *c* (Å)	70.54, 70.54, 168.34	180.18, 180.18, 88.42	54.165, 62.749, 70.557	142.85, 40.458, 76.237
α, β, γ (°)	90, 90, 120	90, 90, 90	90, 105.52, 90	90, 110.55, 90
Resolution range (A)	61.1–1.56 (1.60–1.56)	127.4–2.59 (2.96–2.59)	62.7–1.43 (1.46–1.43)	42.0–1.96 (1.99–1.96)
No. of reflections	652088	352923	272378 (11288)	86249 (4396)
Unique reflections	70030	26817	82986 (4116)	27484 (1445)
Multiplicity	9.3 (6.8)	13.2 (12.9)	3.3 (2.7)	3.1 (3.0)
Completeness (%)	99.9 (99.9)	100.0 (99.9)	99.4 (99.4)	92.0 (99.6)
〈*I*/σ(*I*)〉	19.1 (1.4)	5.0 (1.0)	15.5 (2.2)	9.0 (1.9)
*R*_merge_ (%)	4.8 (101)	3.5 (152)	3.4 (36.0)	12.6 (111.7)
CC_1/2_	0.999 (0.579)	0.978 (0.369)	0.999 (0.992)	0.990 (0.386)
*R*_meas_ (all *I* ^+^ and *I* ^−^)	0.051 (1.182)	0.243 (1.032)	0.043 (0.483)	0.151 (1.360)
Refinement				
Overall Wilson *B* (Å^2^)	30.7	33.3	15.8	24.8
R.m.s. deviations				
Bond lengths (Å)	0.01	0.01	0.01	0.01
Bond angles (°)	1.12	1.23	1.05	1.03
No. of atoms				
Protein	3099	6803	3565	2915
Ligand	0	0	0	20
Water	308	231	656	248
Ligand name [PDB code]	NA	NA	NA	DMSO [DMS]
*B* factors (Å^2^)				
Protein	37.11	36.40	21.26	45.64
Ligand	NA	NA	NA	74.80
Water	47.18	23.01	32.90	42.77
*R*_work_/*R*_free_ (%)	20.4/23.3	18.4/24.4	18.5/21.6	19.3/21.9
Ramachandran parameters				
Preferred (%)	97.0	94.6	96.5	97.5
Allowed (%)	2.5	4.7	1.9	1.9
