# Supplementary material for: Antibody fragments structurally enable a drug-discovery campaign on the cancer target Mcl-1
Source: Acta Crystallogr D Struct Biol. 2019 Oct 31;75(Pt 11):1003–14. doi: 10.1107/S2059798319014116 (PMC6834078; doi:10.1107/S2059798319014116)
Supplement: Supplementary file 1 [file d-75-01003-sup1.pdf]

Acta Cryst D STRUCTURAL BIOLOGY

Volume 75 (2019)

Supporting information for article:

Antibody fragments structurally enable a drug-discovery campaign on the cancer target Mcl-1

Jakub Luptak, Michal Bista, David Fisher, Liz Flavell, Ning Gao, Kate Wickson, Steven L. Kazmirski, Tina Howard, Philip B. Rawlins and David Hargreaves

(A)

```
                1  2  3  4  5  6  7  8  9  10 11 12 13 14 15 16 17 18 19 20 21 22 23 24 25 26 27 28 29 30
scFv (heavy)    Q  V  T  L  K  E  S  G  G  G  L  V  K  P  G  G  S  L  R  L  S  C  A  A  S  G  F  T  F  S

                1  2  3  4  5  6  7  8  9  10 11 12 13 14 15 16 17 18 19 20 21 22 23 24 25 26 27 28 29 30
Fab VH          Q  V  T  L  K  E  S  G  G  G  L  V  K  P  G  G  S  L  R  L  S  C  A  A  S  G  F  T  F  S

                31 32 33 34 35 36 37 38 39 40 41 42 43 44 45 46 47 48 49 50 51 52 53 54 55 56 57 58 59 60
scFv (heavy)    S  Y  S  M  N  W  V  R  Q  A  P  G  K  G  L  E  W  V  S  S  I  S  S  S  S  S  Y  I  Y  Y

                31 32 33 34 35 36 37 38 39 40 41 42 43 44 45 46 47 48 49 50 51 52 53 54 55 56 57 58 59 60
Fab VH          S  Y  S  M  N  W  V  R  Q  A  P  G  K  G  L  E  W  V  S  S  I  S  S  S  S  S  Y  I  Y  Y

                61 62 63 64 65 66 67 68 69 70 71 72 73 74 75 76 77 78 79 80 81 82 83 84 85 86 87 88 89 90
scFv (heavy)    A  D  S  V  K  G  R  F  T  I  S  R  D  N  A  K  N  S  L  Y  L  Q  M  N  S  L  R  A  E  D

                61 62 63 64 65 66 67 68 69 70 71 72 73 74 75 76 77 78 79 80 81 82 83 84 85 86 87 88 89 90
Fab VH          A  D  S  V  K  G  R  F  T  I  S  R  D  N  A  K  N  S  L  Y  L  Q  M  N  S  L  R  A  E  D

                91 92 93 94 95 96 97 98 99 100 101 102 103 104 105 106 107 108 109 110 111 112 113 114 115 116 117
scFv (heavy)    T  A  V  Y  Y  C  A  R  Q  V   G   A   T   W   A   F   D   I   W   G   Q   G   T   L   V   T   V

                91 92 93 94 95 96 97 98 99 100 101 102 103 104 105 106 107 108 109 110 111 112 113 114 115 116 117
Fab VH          T  A  V  Y  Y  C  A  R  Q  V   G   A   T   W   A   F   D   I   W   G   Q   G   T   L   V   T   V

                118 119 120 121 122 123 124 125 126 127 128 129 130 131 132 133 134 135
scFv GS linker  S   S   G   G   G   G   S   G   G   G   G   S   G   G   G   G   S   A

                136 137 138 139 140 141 142 143 144 145 146 147 148 149 150 151 152 153 154 155 156 157 158 159 160 161 162 163 164 165
scFv(light)     Q   S   V   L   T   Q   P   P   S   A   S   G   T   P   G   Q   R   V   T   I   S   C   S   G   S   S   S   N   I   G

                1  2  3  4  5  6  7  8  9  10 11 12 13 14 15 16 17 18 19 20 21 22 23 24 25 26 27 28 29 30
Fab VL          Q  S  V  L  T  Q  P  P  S  A  S  G  T  P  G  Q  R  V  T  I  S  C  S  G  S  S  S  N  I  G

                166 167 168 169 170 171 172 173 174 175 176 177 178 179 180 181 182 183 184 185 186 187 188 189 190 191 192 193 194 195
scFv(light)     S   N   T   V   N   W   Y   Q   Q   L   P   G   T   A   P   K   L   L   I   Y   S   N   N   Q   R   P   S   G   V   P

                31 32 33 34 35 36 37 38 39 40 41 42 43 44 45 46 47 48 49 50 51 52 53 54 55 56 57 58 59 60
Fab VL          S  N  T  V  N  W  Y  Q  Q  L  P  G  T  A  P  K  L  L  I  Y  S  N  N  Q  R  P  S  G  V  P

                196 197 198 199 200 201 202 203 204 205 206 207 208 209 210 211 212 213 214 215 216 217 218 219 220 221 222 223 224 225
scFv(light)     D   R   F   S   G   S   K   S   G   T   S   A   S   L   A   I   S   G   L   Q   S   E   D   E   A   D   Y   Y   C   A

                61 62 63 64 65 66 67 68 69 70 71 72 73 74 75 76 77 78 79 80 81 82 83 84 85 86 87 88 89 90
Fab VL          D  R  F  S  G  S  K  S  G  T  S  A  S  L  A  I  S  G  L  Q  S  E  D  E  A  D  Y  Y  C  A

                226 227 228 229 230 231 232 233 234 235 236 237 238 239 240 241 242 243 244 245 246 247 248 249 250 251 252 253 254 255
scFv(light)     A   W   D   D   S   L   N   A   W   V   F   G   G   G   T   K   L   T   V   L   G   A   A   A   E   N   L   Y   F   Q

                91 92 93 94 95 96 97 98 99 100 101 102 103 104 105 106 107 108 109 110 111
Fab VL          A  W  D  D  S  L  N  A  W  V   F   G   G   G   T   K   L   T   V   L   G

                119 120 121 122 123 124 125 126 127 128 129 130 131 132 133 134 135 136 137 138 139 140 141 142 143 144 145 146 147 148
Fab CH          A   A   K   T   T   P   P   S   V   Y   P   L   A   P   G   S   A   A   Q   T   N   S   M   V   T   L   G   C   L   V

                149 150 151 152 153 154 155 156 157 158 159 160 161 162 163 164 165 166 167 168 169 170 171 172 173 174 175 176 177 178
Fab CH          K   G   Y   F   P   E   P   V   T   V   T   W   N   S   G   S   L   S   S   G   V   H   T   F   P   A   V   L   Q   S

                179 180 181 182 183 184 185 186 187 188 189 190 191 192 193 194 195 196 197 198 199 200 201 202 203 204 205 206 207 208
Fab CH          D   L   Y   T   L   S   S   S   V   T   V   P   S   S   P   R   P   S   E   T   V   T   C   N   V   A   H   P   A   S

                209 210 211 212 213 214 215 216 217 218 219 220 221 222 223 224 225 226 227 228 229 230
Fab CH          S   T   K   V   D   K   K   I   V   P   R   D   C   A   A   A   E   N   L   Y   F   Q

                112 113 114 115 116 117 118 119 120 121 122 123 124 125 126 127 128 129 130 131 132 133 134 135 136 137 138 139 140 141
Fab CL          E   S   E   G   Q   P   K   S   S   P   S   V   T   L   F   P   P   S   S   E   E   L   E   T   N   K   A   T   L   V

                142 143 144 145 146 147 148 149 150 151 152 153 154 155 156 157 158 159 160 161 162 163 164 165 166 167 168 169 170 171
Fab CL          C   T   I   T   D   F   Y   P   G   V   V   T   V   D   W   K   V   D   G   T   P   V   T   Q   G   M   E   T   T   Q

                172 173 174 175 176 177 178 179 180 181 182 183 184 185 186 187 188 189 190 191 192 193 194 195 196 197 198 199 200 201
Fab CL          P   S   K   Q   S   N   N   K   Y   M   A   S   S   Y   L   T   L   T   A   R   A   W   E   R   H   S   S   Y   S   C

                202 203 204 205 206 207 208 209 210 211 212 213 214 215 216 217 218 219 220
Fab CL          Q   V   T   H   E   G   H   T   V   E   K   S   L   S   R   A   D   C   S
```

(B)

```
Chimera   ------------------------------------------------------------   0
Q07820    MFGLKRNAVIGLNLYCGGAGLGAGSGGATRPGGRLLATEKEASARREIGGGEAGAVIGGS   60

Chimera   ------------------------------------------------------------   0
Q07820    AGASPPSTLTPDSRRVARPPPIGAEVPDVTATPARLLFFAPTRRAAPLEEMEAPAADAIM   120

Chimera   --------------------------------------------GPLGSEDDLYRQSLE   15
Q07820    SPEEELDGYEPEPLGKRPAVLPLLELVGESGNNTSTDGSLPSTPPPAEEEEDELYRQSLE   180
                                                          .**:*******

Chimera   IISRYLREQATGSKDSKPLGEAGAAGRRALETLRRVGDGVQRNHETAFQGMLRKLDIKNE   75
Q07820    IISRYLREQATGAKDTKPMGRSGATSRKALETLRRVGDGVQRNHETAFQGMLRKLDIKNE   240
          ************:**:**:*.:**:.*:********************************

Chimera   DDVKSLSRVMIHVFSDGVTNWGRIVTLISFGAFVAKHLKTINQESCIEPLAESITDVLVR   135
Q07820    DDVKSLSRVMIHVFSDGVTNWGRIVTLISFGAFVAKHLKTINQESCIEPLAESITDVLVR   300
          ************************************************************

Chimera   TKRDWLVKQRGWDGFVEFFHVEDLEGG----------------------- 162
Q07820    TKRDWLVKQRGWDGFVEFFHVEDLEGGIRNVLLAFAGVAGVGAGLAYLIR 350
          ***************************
```

Figure S1 (**A**) Table showing alignment and the amino acid numbering for the scFv and Fab. Light shading shows the range of the CDRs according to MOE antibody modeller software (MOE, 2014). Dark shading highlights specific amino acids involved in direct contact with Mcl-1 BH2. (**B**) Alignment of full length Mcl-1 with the human-mouse chimera used in crystallisation. Blue box indicates the mouse sequence.

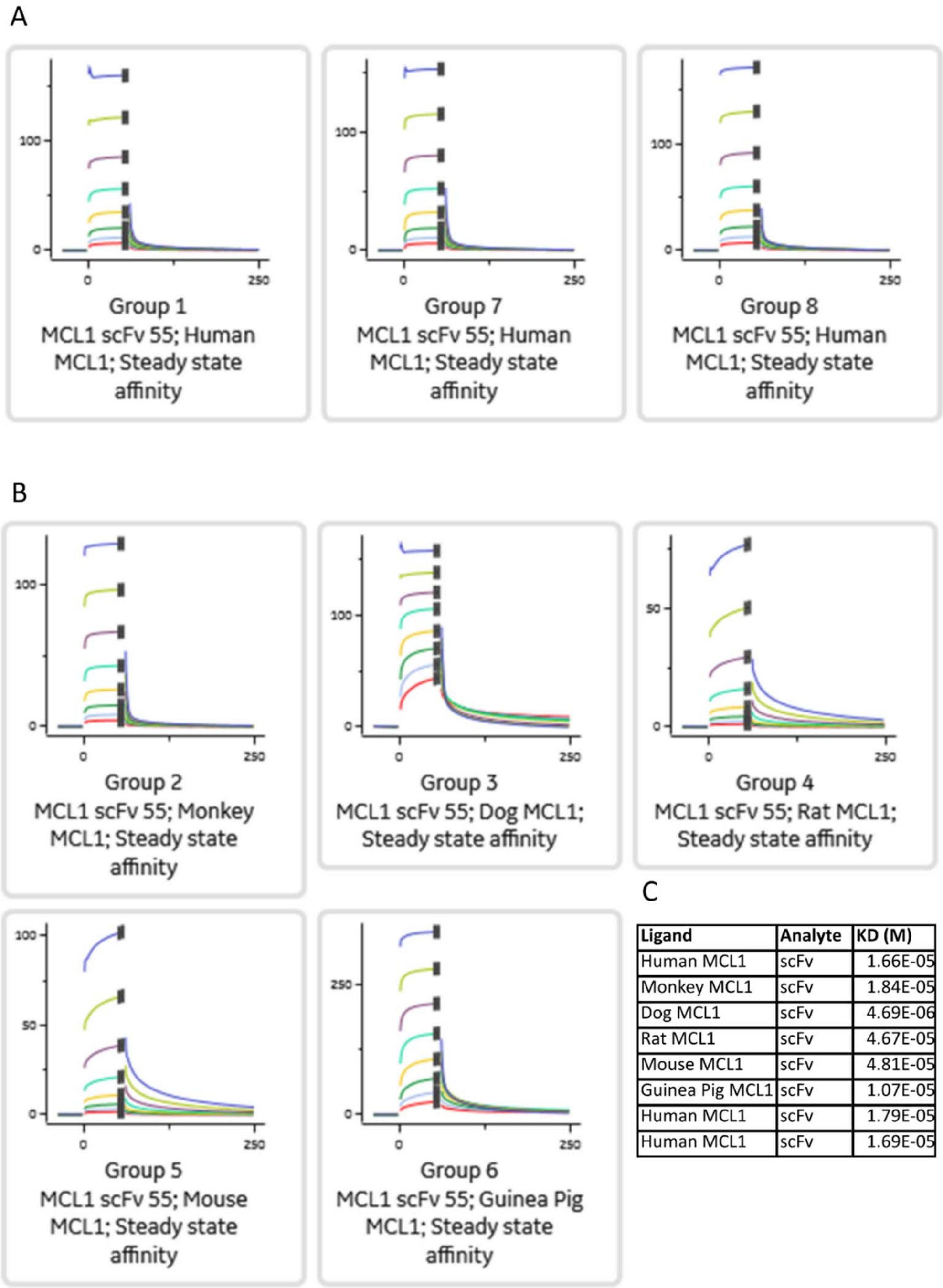

Ligand	Analyte	KD (M)
Human MCL1	scFv	1.66E-05
Monkey MCL1	scFv	1.84E-05
Dog MCL1	scFv	4.69E-06
Rat MCL1	scFv	4.67E-05
Mouse MCL1	scFv	4.81E-05
Guinea Pig MCL1	scFv	1.07E-05
Human MCL1	scFv	1.79E-05
Human MCL1	scFv	1.69E-05

Figure S2 Binding sensorgrams of different orthologues of Mcl-1 immobilised on the SPR chip with scFv injected as an analyte. (**A**) shows three replicates of human Mcl-1, while (**B**) shows different species and their binding response to scfv. (**C**) summarises the steady state K_d obtained. The Groups refer to the 8 channels available on the Biacore 8000.

(A) SPR parameters

Ligand (Mcl-1)	Analyte	Kd (µM)	RU_{imm}	Theoretical R_{max}	R_{max}	Est. surface activity	A-B-A injection with compound AZD5991: Kd (µM)	A-B-A injection with compound AZD5991: Fold Difference
Human	scFv	16.60	445.6	844	218.4	26%	14.2	1.17
Monkey	scFv	18.40	371.5	704	182.6	26%	16.5	1.11
Dog	scFv	46.90	285.4	541	126.1	23%	3.84	1.22
Rat	scFv	46.70	293.4	556	166.9	30%	39.5	1.18
Mouse	scFv	48.10	352.3	668	224.5	34%	39.3	1.22
Guinea	scFv	10.70	855.9	1622	410.9	25%	12.3	0.87
Human	scFv	17.90	435.8	826	214.3	26%	14.5	1.23
Human	scFv	16.90	476.6	903	234.7	26%	16.8	1.00

(B) ITC parameters

Syringe	Sample	N (sites)	Error +/-	K (M^{-1})	Error +/-	Kd (µM)	Error +/-	ΔH (cal/mol)	Error +/-	ΔS (cal/mol/deg)
scFv	Mcl-1	0.92	0.04	2.87E+05	4.57E+04	3.84	0.66	-1.16E+04	6.86E+02	-1.38E+01
scFv	Mcl-1	1.13	0.03	7.90E+05	1.23E+05	1.27	0.23	-9484	349.5	-4.81
Fab	Mcl-1	1.29	0.03	8.77E+05	1.31E+05	1.14	0.20	-6814	243.3	-4.33

(C) DSF parameters

Method	Mcl-1 Tm (°C)	scFv Tm(°C)	scFv Tm(°C) (2 fold excess of Mcl1)	scFv Tm(°C) (10 fold excess of Mcl1)
1st derivative	78.23 ± 0.08	70.53 ± 0.01	71.65 ± 0.01	73.8 ± 0.2

Figure S3 Tables showing biophysical parameters. **(A)** Table lists steady state affinity derived K_d for each of the 8 channels used in the experiment. Ligands denote the immobilised protein, while analyte refers to the injected protein. Ru imm refers to the RU immobilised in each surface, while the theoretical Rmax estimates the extent of response assuming full occupancy of 1:1 binding (Theoretical $R_{max}=R_{imm}*(MW_{analyte}/MW_{ligand})$. R_{max} gives the fitted R_{max} which is then used to calculate the estimated surface activity (R_{max}/Theoretical R_{max}).To perform the ABA injection experiment, AZ5991 had been initially injected at a saturating concentration (1 µM) and the scFv had been titrated in a twofold dilution up to 40 µM. Data were evaluated with Biacore 8K Evaluation Software. Last two columns list K_d for scfv binding with AZD5991 pre-bound with an A-B-A injection. **(B)** ITC parameters. **(C)** DSF parameters.

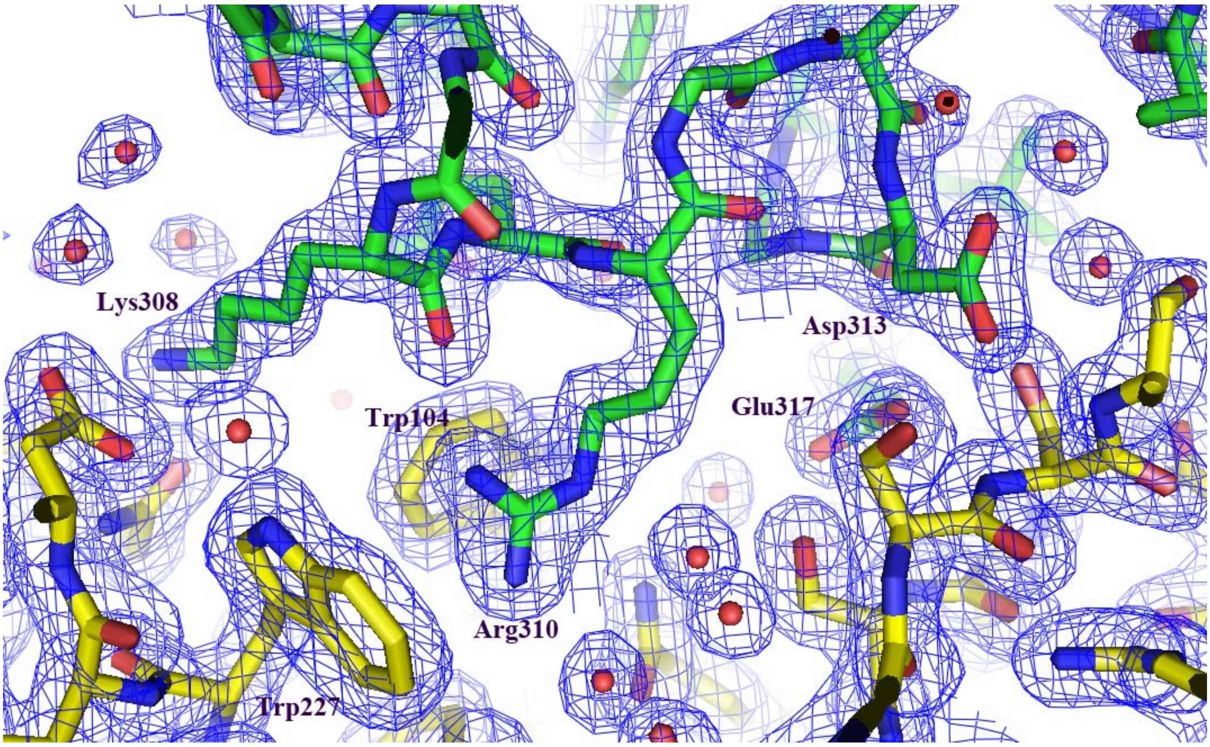

Figure S4 Figure showing an example of the 2Fo-Fc electron density for epitope-paratope interaction between Mcl-1 and the scFv (deposition 6QB3) at 1.9Å. The buried Arginine 310 side chain (green) is shown in the CDR pocket (yellow). Waters are shown as red spheres. Key residues referred to in the main text are labelled.

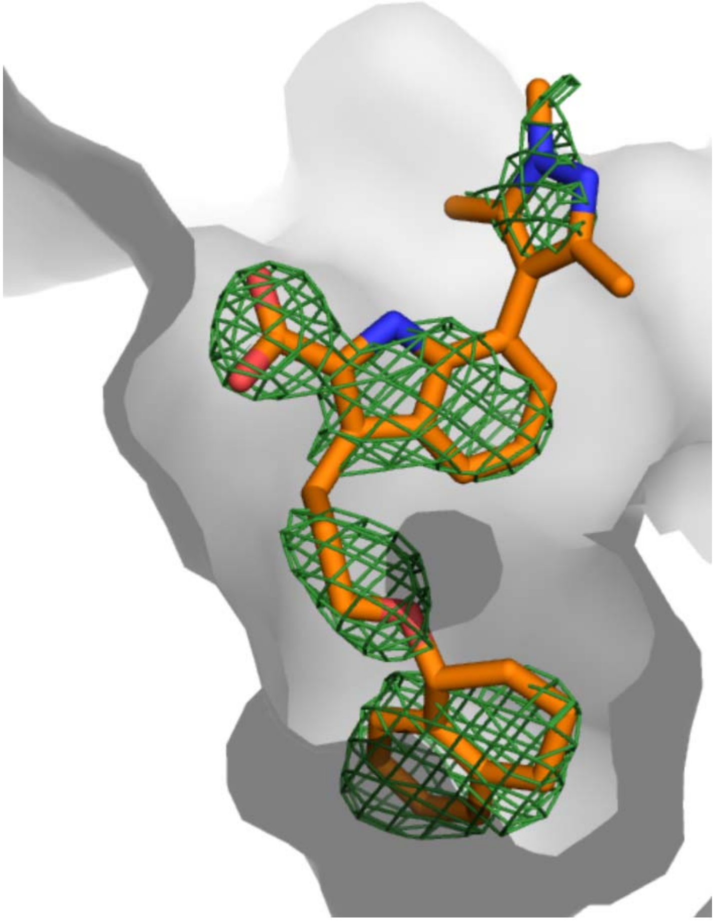

Figure S5 Figure showing a simple omit map of the ligand binding site. The Fo-Fc density was calculated by removing the ligand from the model and re-refining the phases using Buster. The map is contoured at 3σ.

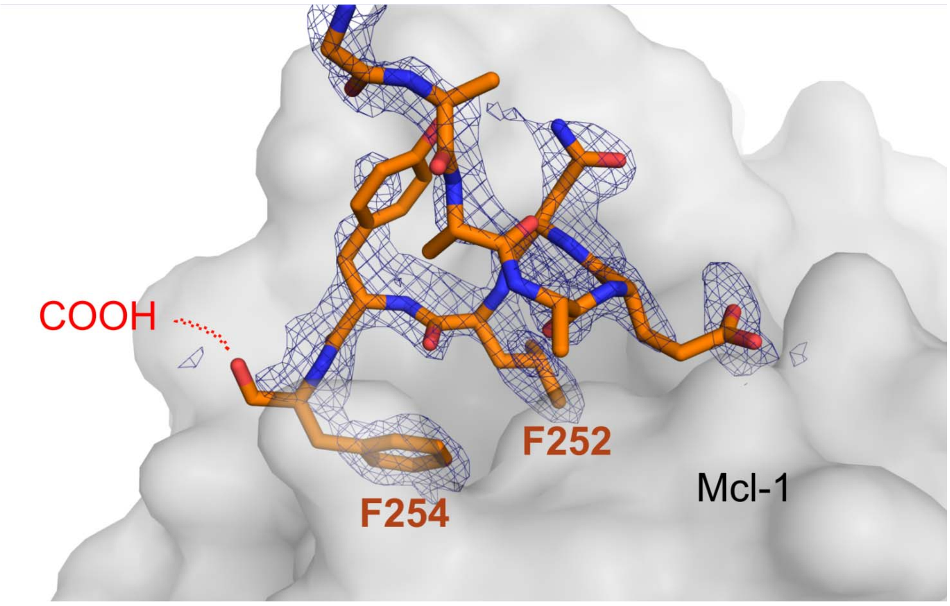

Figure S6 The 2Fo-Fc density was calculated by removing the C-terminal from the model and re-refining the phases using Buster. The map is contoured at 1σ. The carboxy terminal is marked.

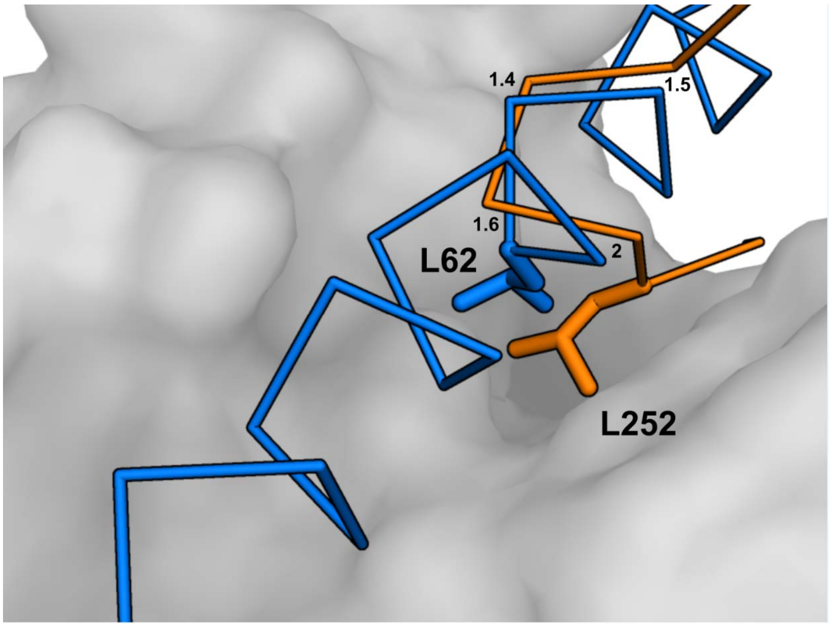

Figure S7 Figure showing and Cα overlay between the C-terminal region of the scFv bound in the BH3 binding site (orange) and the BimBH3 peptide from 2NL9 (blue). Mcl-1 is shown as a surface. Cα distances are shown. The close overlay of L252 and L62 side chains are shown.

References

Molecular Operating Environment (MOE) software. Chemical Computing Group Inc. 2014. http://www.chemcomp.com
